# Biosensors Platform Based on Chitosan/AuNPs/Phthalocyanine Composite Films for the Electrochemical Detection of Catechol. The Role of the Surface Structure

**DOI:** 10.3390/s20072152

**Published:** 2020-04-10

**Authors:** Coral Salvo-Comino, Alfonso González-Gil, Javier Rodriguez-Valentin, Celia Garcia-Hernandez, Fernando Martin-Pedrosa, Cristina Garcia-Cabezon, Maria Luz Rodriguez-Mendez

**Affiliations:** 1 Group UVASENS, Escuela de Ingenierías Industriales, Universidad de Valladolid, Paseo del Cauce, 59, 47011 Valladolid, Spain; coraldeugena@hotmail.com (C.S.-C.); alfonsogoninf@gmail.com (A.G.-G.); javrova@gmail.com (J.R.-V.); celiagarciahernandez@gmail.com (C.G.-H.); 2 Bioeco UVA Research Institute, Universidad de Valladolid, 47011 Valladolid, Spaincrigar@eii.uva.es (C.G.-C.); 3 Dpt. of Materials Science, Universidad de Valladolid, Paseo del Cauce, 59, 47011 Valladolid, Spain

**Keywords:** catechol, gold nanoparticles, sulfonated phthalocyanine, laccase, tyrosinase, layer-by-layer

## Abstract

Biosensor platforms consisting of layer by layer films combining materials with different functionalities have been developed and used to obtain improved catechol biosensors. Tyrosinase (Tyr) or laccase (Lac) were deposited onto LbL films formed by layers of a cationic linker (chitosan, CHI) alternating with layers of anionic electrocatalytic materials (sulfonated copper phthalocyanine, CuPcS or gold nanoparticles, AuNP). Films with different layer structures were successfully formed. Characterization of surface roughness and porosity was carried out using AFM. Electrochemical responses towards catechol showed that the LbL composites efficiently improved the electron transfer path between Tyr or Lac and the electrode surface, producing an increase in the intensity over the response in the absence of the LbL platform. LbL structures with higher roughness and pore size facilitated the diffusion of catechol, resulting in lower LODs. The [(CHI)-(AuNP)-(CHI)-(CuPcS)]_2_-Tyr showed an LOD of 8.55∙10^−4^ μM, which was one order of magnitude lower than the 9.55·10^−3^ µM obtained with [(CHI)-(CuPcS)-(CHI)-(AuNP)]_2_-Tyr, and two orders of magnitude lower than the obtained with other nanostructured platforms. It can be concluded that the combination of adequate materials with complementary activity and the control of the structure of the platform is an excellent strategy to obtain biosensors with improved performances.

## 1. Introduction

The accurate detection of phenolic compounds has attracted great attention due to their wide use in different applications; these include their use as antioxidants in health-care, pharmacology or agriculture among others. They are also used as pesticides and are byproducts of many industries [1]. So far, several analytical methods have been developed for catechol detection, including electrochemical biosensing. The appropriate selection of the immobilization surface is of great importance to develop biosensors with enhanced sensing performance. The layer-by-layer (LbL) technique is a suitable alternative to immobilize enzymes without affecting their activity [2,3,4,5]. In addition, LbL films show a high versatility for designing films with different compositions and functionalities with controlled architecture. They have been used to incorporate a variety of electrocatalytic materials that can improve the electronic communication between the biomolecule and the electrode substrate. 

Owing to their high specific surface area and excellent electrocatalytic properties, nanomaterials such as graphene or metallic nanoparticles have been widely used to improve the performance of biosensors dedicated to the detection of phenols [6,7,8,9,10]. Other electrocatalysts, such as conducting polymers [11,12], porphyrins [13,14] or phthalocyanines [15,16,17], have also been successfully incorporated into LbL films as electrocatalytic materials in electrochemical biosensors. On the other hand, LbL films are ideal structures to incorporate linkers able to capture the enzymatic probes. Amongst the different linkers, researchers have paid particular attention to chitosan, which has shown excellent properties for enzyme immobilization in electrochemical biosensors [18,19,20].

The performance of biosensors can be further improved by combining materials with different functionalities. For instance, electrocatalytic materials (i.e CNT) have been combined with enzyme linkers (chitosan (CHI), polyanilline (PAH), dipalmitoylphosphatidylglycerol (DPPG), etc.) [21]. In another strategy, electrocatalytic materials with complementary activity have been combined in order to produce synergistic effects. Some examples of effective combinations include mixtures of conducting polymers with AuNPs [22], phthalocyanines with AuNPs [23,24,25,26], graphene with porphyrine [27] or graphene with metal oxides [28].

In a previous work, our group explored the possibility of using the LbL technique to build up layers of three materials with complementary activity: an electron mediator (copper phthalocyanine), an enzyme linker (CHI) and an ionic liquid (IL) whose function was to increase the conductivity of the surface [29]. The results showed an increase in the sensitivity, however the improvement in the limit of detection was small. The main objective of this work was to improve the limit of detection of a catechol biosensor detection by combining an enzyme linker with two electrocatalytic materials. The combination of two electron mediators could improve the sensitivity of the sensors due to synergistic effects.

On the other hand, the structure of the underlying platform can have an influence on the efficiency of the electron transfer between the enzymatic redox-active site and the electrode surface. In this sense, the LbL technique can be used to prepare films with different arrangements and this makes this technique a valuable tool to analyze the role of the surface. For this reason, a secondary objective of this work was to evaluate the effect of the surface structure and porosity of the surface of the platforms. 

To achieve these objectives, in this work LbL films have been used as biosensor platforms for the immobilization of tyrosinase and laccase (two copper-containing enzymes that catalyze the conversion of phenols to highly reactive quinones). Biosensing platforms have been obtained by combining a cationic material acting as enzyme linker (CHI) with two anionic electron mediators including gold nanoparticles (AuNP) and a sulfonated copper phthalocyanine (CuPcS). The improvement of the performance caused by the combination of materials and the possible existence of synergistic effects has been evaluated by analyzing the sensitivity, linear range, limits of detection and stability of the biosensors in the detection of catechol. The performance of LbL-based biosensors with different structures (variable arrangement in the sensor components) and surface roughness has also been discussed.

## 2. Materials and Methods

### 2.1. Reagents

Chitosan (CHI) (deacetylation degree 75–85%, CAS Number: 9012-76-4), copper(II) phthalo-cyanine-tetrasulfonic acid tetrasodium salt (CuPcS) (dye content 85%), tetrachloroauric(III) acid trihydrate (purity 99.995%), catechol and hydroquinone (purity 99%), lacase (Lac) from *Trametes versicolor* (enzymatic activity 10 U/mg), tyrosinase (Tyr) from *Agaricus bisporus* (enzymatic activity 1000 U/mg) and trisodium citrate dihydrate (purity 99%, CAS Number: 6132-04-3) were purchased from Sigma Aldrich (Saint Louis, MO, USA), as were the chemicals used to prepare buffer solutions. These included sodium dihydrogen phosphate (purity 99%, CAS Number: 7558-79-4) and sodium hydrogen phosphate (purity 99%, CAS Number: 7558-80-7). Acetic acid (purity 99.7%) and acetone (99%) were acquired from Panreac (Barcelona, Spain) and glutaraldehyde (50% in water) was purchased from Alfa Aesar (Tewksbury, MA, USA). Ultrapure water (18.2 MΩ.cm and pH 5.6) was acquired from a Milli-Q system (Millipore Merk, MO, USA).

### 2.2. LbL Film Platforms 

AuNPs colloid was obtained by citrate reduction of tetrachloroauric(III) acid trihydrate following the method proposed by Brust et al. [30]. The CHI(+) solution was prepared by dissolving chitosan 10^−3^ M in acetic acid 0.3% V/V. A 5·10^−5^ M CuPcS(−) solution was prepared using deionized water. 

The LbL films were made by successive immersions of the substrate into the corresponding solutions containing CHI(+), CuPcS(-) and AuNP(−) (Figure S1). Prior to use, ITO glass was carefully cleaned with acetone. The ITO was sonicated in ethanol and deionized water, respectively, after which the electrode was dried under natural air conditions.

Films with different architectures were developed according to the following sequence:

(1) Film sequence [(CHI)-(AuNPs)-(CHI)-(CuPcS)]_2_: CHI solution (5 min) → ultrapure water gently stirred to remove excess of adsorbed CHI (1 min) → AuNPs (5 min)→ ultrapure water gently stirred to remove excess of adsorbed AuNPs → CHI solution (5 min) → Ultrapure water gently stirred to remove excess of adsorbed CHI (1 min) → CuPcS (5 min)→ ultrapure water gently stirred to remove excess of adsorbed CuPcS (1 min).
(2) Film sequence [(CHI)-(CuPcS)-(CHI)-(AuNPs)]_2_: CHI solution (5 min) → ultrapure water gently stirred to remove excess of adsorbed CHI (1 min) → CuPcS (5 min) → ultrapure water gently stirred to remove excess of adsorbed CuPcS (1 min) → CHI solution (5 min) → ultrapure water gently stirred to remove excess of adsorbed CHI (1 min) → AuNPs (5 min)→ ultrapure water gently stirred to remove excess of adsorbed AuNPs (1 min). In all cases, multilayered LbL films were grown, repeating the “four-step sequence” twice. Films with the sequences [(CHI)-(AuNPs)]_2_ or [(CHI)-(CuPcS)]_2_ were also prepared for comparison purposes following the same methodology.

### 2.3. Preparation of Biosensors LbL-Tyr or LbL-Lac

LbL-Tyr and LbL-Lac biosensors were prepared by depositing 50 µL of a 5 mg.mL^−1^ Tyr or Lac suspension in phosphate buffer (0.01 M, pH 7) onto the LbL platforms described above and dried at room temperature. After drying, the films were exposed to a 2.5% (*v/v*) glutaraldehyde solution or vapors (in phosphate buffer 0.01 M, pH 7) for 20 min at room temperature. The LbL enzyme film was dried at 20 °C and rinsed with phosphate buffer solution in order to remove any unbound enzyme from the LbL film surface.

The biosensors made using the above steps were named as follows:

- Biosensors on film sequence 1: [(CHI)-(AuNPs)-(CHI)-(CuPcS)]_2_–Tyr; [(CHI)-(AuNPs)-(CHI)-(CuPcS)]_2_ –Lac_._
- Biosensors on film sequence 2: [(CHI)-(CuPcS)-(CHI)-(AuNPs)]_2_–Tyr; [(CHI)-(CuPcS)-(CHI)-(AuNPs)]_2_ –Lac.

### 2.4. Characterization Techniques

Layer by layer films were deposited onto different substrates (ZnS, quartz and ITO glass) using a rotary dip-coater device ND-R from Nadetech (Pamplona, Spain). UV-Vis spectra were registered using films deposited on quartz substrates with a UV-2600 instrument Shimadzu (Kioto, Japan). Fourier Transform Infrared (FTIR) spectra of films deposited on ZnS were obtained from 700 to 4000 cm^−1^, at a resolution of 4 cm ^-1^ and 1000 scans using a FTIR 6600 spectrophotometer Jasco (Easton, MD, USA). Atomic force microscope (AFM) images were recorded in films deposited on ITO glass at room temperature on a Cypher ES (Asylum Instruments, Santa Barbara, CA, USA) operated in tapping mode and using BlueDrive Photothermal Excitation. Cyclic voltammetry (CV) was conducted using an PGSTAT128 (Autolab Metrohm, Utrecht, The Netherlands) potentiostat/galvanostat with a conventional three-electrode cell. The reference electrode was an Ag|AgCl/KCl saturated electrode and the counter electrode was a Pt plate. Cyclic voltammograms were recorded with a scan rate at 0.1 V∙s^−1^ in the potential range of −0.8 to 1.2 V.

## 3. Results and Discussion

### 3.1. Preparation and Spectroscopic Characterization of LbL Film Platforms

**Figure 1:**
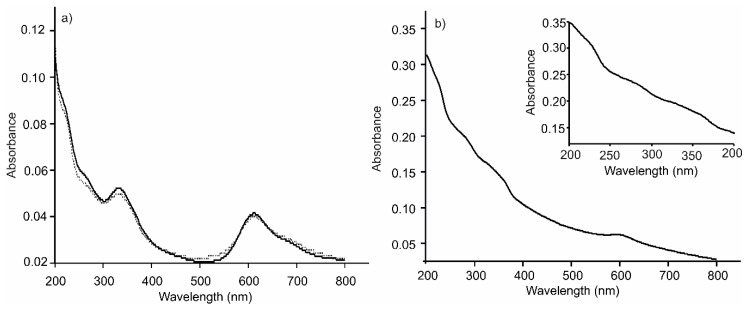
UV-Vis absorption spectra of LbL films of (**a**) [(CHI)-(CuPcS)-(CHI)-(AuNPs)]_2_ (dotted line) and [(CHI)-(AuNPs)-(CHI)-(CuPcS)]_2_ (solid line); (**b**) [(CHI)-(CuPcS)-(CHI)-(AuNPs)]_2_-Tyr. Inset shows a magnification of the band associated to the enzyme.

Multilayer thin films were prepared by the assembly of CHI(+), AuNPs(+) and CuPcS(−), following two different sequences: [(CHI)-(AuNPs)-(CHI)-(CuPcS)]_2_ and [(CHI)-(CuPcS)-(CHI)-(AuNPs)]_2_. The LbL films were characterized in terms of growth using UV-Vis and FTIR absorption spectroscopies. The UV-Vis spectra of the layered structures showed the bands associated to all three components (Figure 1): a band at 280 nm produced by CHI [31] a very weak band at 533 nm produced by the phonons of the AuNPs of 54 nm size [32] (Figure S2) and the typical Q and Soret bands (at 615 and 330 nm respectively) originated by π-π transitions in the phthalocyanine ring [33]. The shoulder observed at 680 nm was produced by the presence of H-aggregates due to interactions between the phthalocyanine rings. Spectra were similar for all the films, regardless of their architecture. According to the UV-Vis spectra, the LbL films grew uniformly in terms of the amount of material per deposited layer (Figure S3).

Once good-quality LbL substrates were obtained, Tyr or Lac was immobilized on the platform surface. As observed in Figure 1b, where the UV-Vis spectra of the [(CHI)-(CuPcS)-(CHI)-(AuNPs)]_2_–Tyr biosensor is shown, biosensors showed the characteristic bands of the LbL platform and the typical band at 280 nm produced by the presence of the enzyme. UV-Vis spectra of the biosensors immersed in phosphate buffer were registered periodically and no changes were observed during 24 h, confirming the appropriate immobilization of the biomolecule. 

FTIR spectra of the LbL platforms deposited on zinc sulfide (ZnS) were also recorded. They displayed the expected vibrational modes of the three components present in the layers: CHI (3385 cm^−1^, 1667 cm^−1^, 1422 cm^−1^ and 1397 cm^−1^); CuPcS (1457 cm^−1^, 1335 cm^−1^, 1149 cm^−1^ and 1033 cm^−1^) and the COO stretch of the citrate at 1720 cm^−1^ (Figure S4) [16,34]. Again, FTIR spectra were identical, regardless of the structure of the LbL films. FTIR also confirmed that layers were deposited homogeneously and transmittance increased linearly with the number of layers (Figure S5).

As expected, the FTIR spectra of the biosensors showed the typical enzymatic amide bands and hydroxyl group that appeared at 1612 cm^−1^ and 1567 cm^−1^ and 3352 cm^−1^ respectively, confirming the adsorption of the enzyme onto the surface of the LbL platform [35].

### 3.2. Surface Characterization

**Figure 2:**
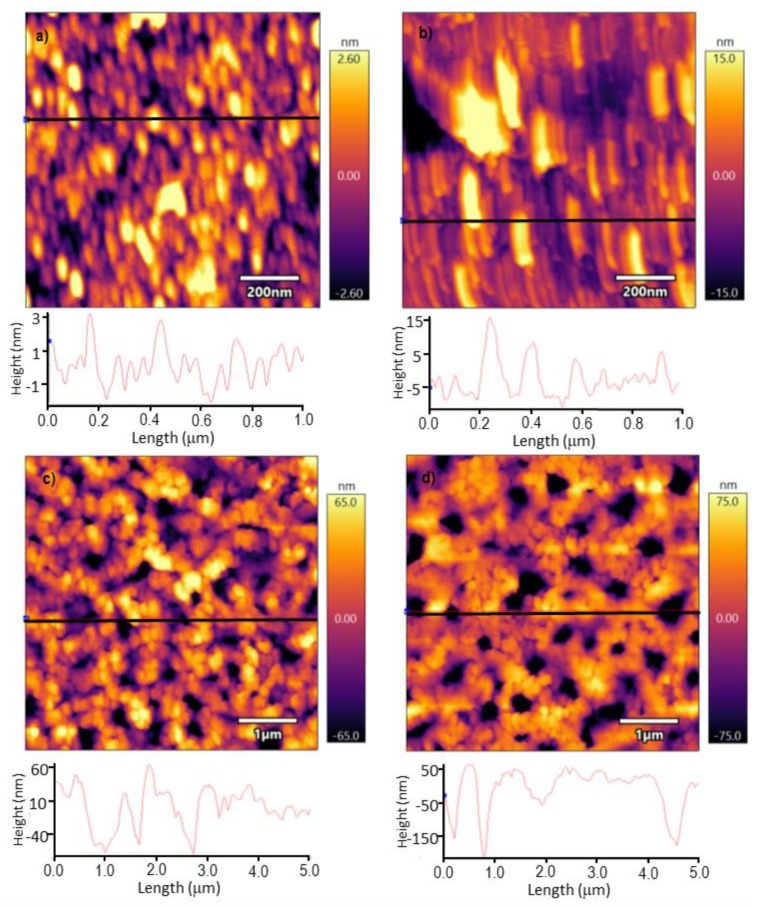
AFM topographic images of (**a**) [(CHI)-(CuPcS)-(CHI)-(AuNPs)]_2_, (**b**) [(CHI)-(AuNPs)-(CHI)-(CuPcS)]_2_; (**c**) [(CHI)-(CuPcS)-(CHI)-(AuNPs)]_2_–Tyr and (**d**) [(CHI)-(AuNPs)-(CHI)-(CuPcS)]_2_–Tyr.

The topography of the films was analyzed by AFM. The topography of the [(CHI)-(CuPcS)-(CHI)-(AuNPs)]_2_ film, where AuNPs were deposited in the surface layer (Figure 2a), showed quasi-spherical structures with an average radius of 40 nm. These data fit well with the diameter of the AuNPs calculated from the UV-Vis spectra [25]. The calculated mean roughness RMS was 1.301 nm. The topography of the [(CHI)-(AuNPs)-(CHI)-(CuPcS)]_2_ film, with phthalocyanine deposited in the last layer (Figure 2b), showed a distinct surface with parallel lamellar structures of 165 nm length and 30 nm width (average values), formed by aggregates of phthalocyanines. The roughness of the surface was clearly higher than that observed when the AuNPs were in the last layer (RMS: 2.5 nm). 

When enzymes were deposited on the top of the LbL platforms, the topography changed and globular enzymatic structures accompanied by wide channels were observed (Figure 2c,d). The depth of the channels and the roughness were strongly influenced by the nature of the subjacent LbL film. When AuNPs formed the last layer and the enzyme was deposited on the top, the depth of the observed channels was 40 nm (RMS 31.862 nm); whereas the depth of the channels formed by the enzyme deposited on the top of CuPcS was 170 nm (RMS: 41.763 nm). The increase in roughness and depth of the channels observed in the [(CHI)-(AuNPs)-(CHI)-(CuPcS)]_2_ platforms resulted in an increased electrochemical intensity (electrochemical analysis by CV to be presented later on), and consequently, at a low limit of detection for analysis.

### 3.3. Electrochemical Characterization of the LbL-Based Biosensors

The electrochemical behavior of catechol on ITO modified LbL films was studied using cyclic voltammetry. Then, the LbL films were used as platforms for enzyme immobilization and their sensing performance was evaluated. CV curves were conducted at a scan rate of 0.1 V.s^−1^ under the potential from −0.8 to 1.2 V.

The response of catechol 10 ^4^ M (in 0.01 M phosphate buffer, pH 7) at a bare ITO electrode was characterized by one weak anodic wave at 1050 mV, which corresponded to the oxidation of the catechol to the quinoid form, and a weak reverse wave at −65 mV with a current density of 12 µA/cm^2^ (Figure S6). These features reveal that the activity of bare ITO is very low for the electrochemical oxidation of catechol. 

**Figure 3:**
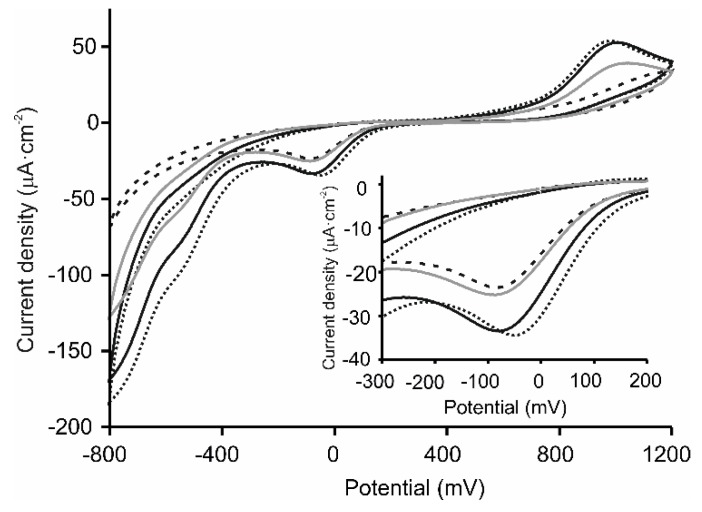
CV curves registered in catechol 10^−4^ mol∙L^−1^ in 0.01 M phosphate buffer pH 7 as electrolyte at [(CHI)-(AuNPs)]_2_ (dashed line), [(CHI)-(CuPcS)]_2_ (solid grey line), [(CHI)-(CuPcS)-(CHI)-(AuNPs)]_2_ (solid line), (CHI)-(AuNPs)-(CHI)-(CuPcS)]_2_ (dotted line) electrodes at a scan rate of 0.1 V∙s^−1^ . The inset shows a magnification of the cathodic peak.

The voltammetric response of successive layers forming the LbL films was registered step by step. As seen in Figure 3, catechol on the LbL platforms produced the already mentioned peaks corresponding to the two-electron oxidation (at 980 mV) and two electron reduction (at –82 mV) of catechol. The current density increased when the ITO glass was covered with layers of the electrocatalytic materials. For instance, the current density of the cathodic wave increased from 12 µA/cm^2^ (bare ITO) to 23 µA/cm^2^ when the ITO was covered with layers of [(CHI)-(AuNPs)]_2_ or [(CHI)-(CuPcS)]_2_. The current density was further increased when both electrocatalytic materials were combined in the same LbL platform. As observed in the figure, the current density for the cathodic wave reached a value of 35 µA/cm^2^, pointing to the existence of synergistic effects between AuNPs and CuPcS. In addition, as discussed above, the modification of the ITO glass with the LbL platforms increased the surface roughness, resulting in a higher number of active sites for catechol. The roughness was higher when the CuPcS was placed in the last layer. Therefore, the response signals of catechol improved on the [(CHI)-(AuNPs)-(CHI)-(CuPcS)]_2_ surface. It is worth noting that, in good accordance with the literature, the influence of the thickness of the LbL films in electrochemical responses was negligible and similar results were obtained using films with a higher number of layers [36].

The next step in this work was to cover the LbL platforms with two different phenol oxidases, tyrosinase (Tyr) and laccase (Lac). Even if π−π interactions between the enzyme and the phthalocyanine aromatic rings can improve the immobilization of the enzymes on the LbL platform, Tyr or Lac were fixed to the LbL platform by cross-linking with glutaradehyde using two different approaches. The first approach consisted in immersing the LbL-Tyr or LbL-Lac in a glutaraldehyde solution. In the second approach, the LbL-enzyme biosensors were exposed to glutaraldehyde vapors. The results obtained showed that the enzymatic activity was better preserved when glutaraldehyde vapors were used for the cross-linking (Figure S7). The responses were highly reproducible and the variation coefficient calculated from five consecutive cycles was 0.79%. For this reason, this immobilization methodology was used in the following experiments.

**Figure 4:**
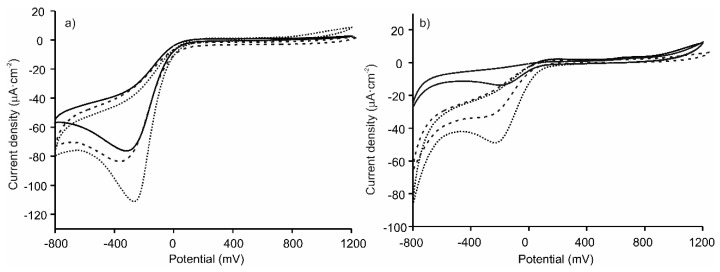
Voltammetric response of catechol 10-4 M in 0.01 M phosphate buffer pH 7 at an (**a**) ITO-Tyr (solid line), [(CHI)-(CuPcS)-(CHI)-( AuNPs)]_2_-Tyr (dashed line), [(CHI)-(AuNPs)-(CHI)-(CuPcS)]_2_-Tyr (dotted line); and (**b**) ITO-Lac (solid line), [(CHI)-(CuPcS)-(CHI)-(AuNPs)]_2_-Lac (dashed line), [(CHI)-(AuNPs)-(CHI)-(CuPcS)]_2_-Lac (dotted line).

Figure 4 compares the responses of ITO-Tyr and ITO-Lac to the responses of the LbL platforms covered with tyrosinase or laccase. As can be seen, catechol can be oxidized at inert electrodes covered with the enzymes. Voltammograms gave a well-defined peak located at –270 mV, which was due to the reduction of o-quinone species formed from the enzymatic reaction at the electrode surface. As the results show, the peak currents of catechol increased drastically when the enzyme was immobilized on the surface of the LbL platforms, indicating that the layered composite plays a key role in enhancing the electrochemical performance. Both types of platforms, [(CHI)-(AuNPs)-(CHI)-(CuPcS)]_2_ and [(CHI)-(CuPcS)-(CHI)-(AuNPs)]_2_, were efficient promoters of the enzymatic activity, yet the overall performance clearly improved when the last layer in contact with the enzyme was a CuPcS layer. In addition, as shown in Figure 2, the [(CHI)-(AuNPs)-(CHI)-(CuPcS)]_2_-Tyr (or Lac) biosensor showed a larger pore size (depth of 170 nm) than the [(CHI)-(CuPcS)-(CHI)-(AuNPs)]_2_-Tyr (or Lac) biosensor (depth of pores 40 nm). Consequently, the catechol molecule (Unit cell: a = 9.732(3)Å, b = 5.620(2)Å, c = 10.332(3)Å [37] could diffuse more easily inside the pores of the electrode surface and reach a higher number of active sites.

One of the objectives of this work was to evaluate the electron mediator capability of the LbL films in the presence of different phenol oxidases. Tyrosinase oxidizes monophenols and o-diphenols to the corresponding quinone; whereas laccase is able to catalyze the one-electron oxidation of several aromatic substrates including *o*-, *p*- and polyphenols. The low specificity of laccase makes it an attractive catalyst for industrial uses [38].

The LbL surface enables both redox enzymes to transfer electrons efficiently to the electrode. In both cases, the best results were obtained when CuPcS was present in the last layer. Although the intensity of the catechol peak increased at the tyrosinase electrode from 72 µA/cm^2^ to 112 µA/cm^2^; in the case of the laccase sensor, the presence of the LbL platform produced a 3-fold increase in the intensity over the response of the biosensor without the LbL platform.

The limits of detection (LOD) were calculated from chronoamperograms registered in solutions with increasing concentrations of catechol (from 2.43 × 10^−6^ to 2.45 x10^−5^ mol∙L^−1^) according to IUPAC, the 3σ/S criterion in which σ is the standard deviation of the response of 5 blanks and S is the sensitivity. Calibration curves were constructed by plotting I_a_ (or I_c_) versus the catechol concentration. The analysis for catechol detection was made by three measurements for each architecture sensor to obtain the LOD results and verify the reproducibility for the sensors.

**Figure 5:**
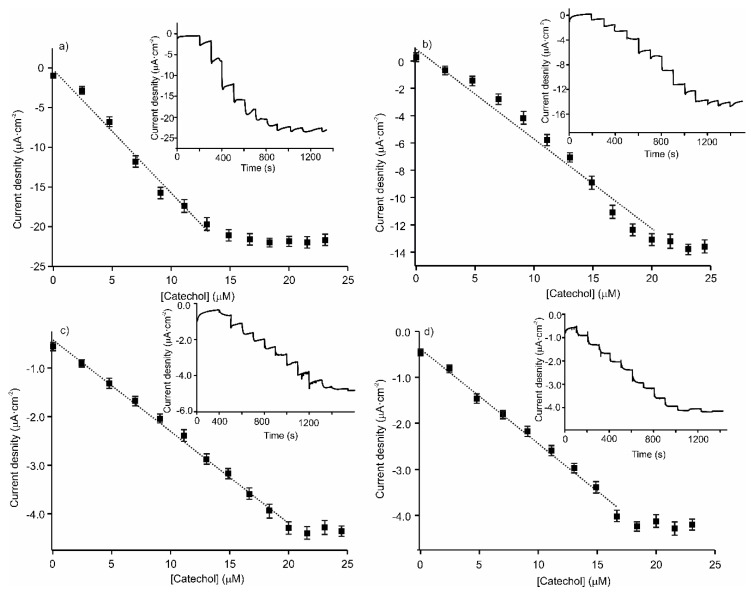
Calibration curves of the amperometric response for increasing the concentration of catechol at (**a**) [(CHI)-(CuPcS)-(CHI)-(AuNPs)]_2_-Tyr; (**b**) [(CHI)-(AuNPs)-(CHI)-(CuPcS)]_2_-Tyr; (**c**) [(CHI)-(CuPcS)-(CHI)-(AuNPs)]_2_-Lac; and (**d**) [(CHI)-(AuNPs)-(CHI)-(CuPcS)]_2_-Lac. The insets show the amperometric responses obtained for each biosensor.

The peak currents, from which the sensitivity and LODs were determined, increased with the catechol concentration. A linear response was observed in the 2.4–20 µM range, with a higher sensitivity for the tyrosinase-based biosensor (Figure 5). Sensitivity and LODs were calculated from the linear parts of those plots (Table 1). The LODs were lower, or in the range of those reported for biosensors based on tyrosinase or laccase containing LbL films phthalocyanines as electron mediator, confirming the improvement in the electron transfer provided by the LbL platform.

As is well known, the sensitivity and the LOD depend on the accessible surface area of the working electrode [39]. In our case, the direct interaction LbL-enzyme was higher when the roughness of the LbL layer increased. For instance, the LOD obtained for [(CHI)-(CuPcS)-(CHI)-(AuNPs)]-Tyr was one order of magnitude lower than that obtained for [(CHI)-(AuNPs)-(CHI)-(CuPcS)]-Tyr. In short, the roughness of the LbL platforms, combined with the electrocatalytic properties of the materials forming the layers, improved the electrochemical performance of the biosensors.

Table 1 collects data obtained with similar biosensors using platforms where materials similar to those used in this work were present. As observed in the Table, the use of the LbL technique, the combination of appropriate materials and the control of the porosity of the platform surface, have allowed us to improve the LODs obtained in previous research. Representing 1/I_lim_ vs. 1/[S_ox_] (according to the Linewaever-Burk approach), the apparent Michaelis-Menten constant was obtained (Table 1). The $K_{M}^{app}$ obtained were lower than those reported for the free enzyme (2.30 mM as reported by [39] or by other biosensors where enzymes were deposited on LbL or LB films (reported in Table 1). This high activity could be associated to the presence of the LbL platform, which promotes the electron transfer while providing a high porosity where catechol can diffuse and active sites are adequately exposed.

### 3.4. Reproducibility and Repeatability

The reproducibility of the LbL-based biosensors was investigated. It was estimated from the response to 10^−4^ M catechol by three modified electrodes prepared under the same conditions. A relative standard deviation (RSD) of 8.71% was obtained. The RSD of the amperometric response to 10^−4^ M catechol was 1.07% for 3 successive measurements, indicating the good repeatability.

## 4. Conclusions

In this study, we describe the use of a layer-by-layer film of a combination of electrocatalytic materials with complementary activity (chitosan, sulfonic copper phthalocyanine and gold nanoparticles) to immobilize the phenol oxidases enzymes tyrosinase and laccase efficiently. The LbL technique has been shown to be an efficient method for developing sensing platforms, as it allows an intimate contact between materials, facilitating interactions that promote the existence of synergistic effects. In addition, the effective combination of materials in LbL platforms brings other advantages in electrochemical detection, including the efficient transmission of charges, higher roughness and porosity for adsorption and transport, and more sensing sites; thus, the developed [(CHI)-(AuNP)-(CHI)-(CuPcS)]_2_ films have been demonstrated to be excellent platforms for biosensing. The control of the structure and surface of the LbL platform has allowed us to decrease the LOD obtained, in comparison with other platforms, by two orders of magnitude.

## Figures and Tables

**Table 1:** Analytical parameters of biosensors combining similar materials.

Biosensor Description	R ^2^	Sensitivity (A M^−1^)	LOD (µM)	Linear Range (µM)	$K_{M}^{app}\;(µM)$	Ref.
** [(CHI)-( CuPcS)-(CHI)-( AuNPs)]-Tyr**	0.969	1.500	9.55·10^−3^	2.4–14.9	11.04	This work
** [(CHI)-(AuNPs)-(CHI)-(CuPcS)]-Tyr**	0.964	0.681	8.55·10^−4^	2.4–20.0	14.41	This work
** [(CHI)-( CuPcS)-(CHI)-( AuNPs)]-Lac**	0.991	0.384	5.89·10^−2^	2.4–16.6	8.76	This work
** [(CHI)-(AuNPs)-(CHI)-(CuPcS)]-Lac**	0.996	0.188	1.84·10^−2^	2.4–20.0	7.45	This work
** Tyr-AuNP-SPCE**	--	0.08	0.2	0.8–120.0	--	[7]
** [CHI**^** (+)**^** +IL**^** (+)**^** |CuPc**^** S(-)**^]_** 2**_** |Lac**	0.981	0.230	9.98·10^−3^	2.4–14.9	3.16	[29]
** Tyr-AuNPs-SPCE**	0.993	0.55	1.2	2.5–20.0	--	[39]
** CoPc-CGCE-Tyr**	0.991	0.160	4.50·10^−1^	5.0–1000.0	1600.00	[40]
** Lac/MWCNT/AuNPs-SDBS-PEDOT/GCE**	0.960	0.012	1.10·10^−1^	0.1–0.5	--	[41]
** Lac/AA/LuPc**_** 2**_	0.992	--	4.88·10^−1^	4.0–150.0	--	[42]
** Tyr/AA/LuPc**_** 2**_	0.997	--	5.18·10^−1^	4.0–150.0	--	[42]
** Tyr/AA/LuPc**_** 2**_	--	--	1.71	1.9–27.5	63.72	[43]
** CoPc-CPEs-Tyr**	--	0.002	7.5	30.0–320.0	120.00	[44]
** Tyr-magnetite-CHI-GCE**	0.996	0.057	2.62·10^−1^	1.0–30.0	22.5	[45]
** MWCNT/Lac/CHI-CPE**	0.999	0.279	3.34·10^−2^	0.1–165.0	--	[46]

## Supplementary Materials

The following are available online at https://www.mdpi.com/1424-8220/20/7/2152/s1, Figure S1: Materials used to form the sensing platforms: a) gold nanoparticles (AuNPs) capped with citrate; b) sulfonated copper phthalocyanine (CuPcS); and c) chitosan (CHI), Figure S2: UV-Vis spectrum of AuNP solution, Figure S3: UV-Vis spectra of a) [(CHI)-(AuNPs)-(CHI)-(CuPcS)]_2_ LbL films with increasing number of layers (n = 4,8,12,16,20); and b) correlation between the absorbance measured at 615 nm and the number of layers, Figure S4: Comparison of the FTIR spectra of the LbL platform [(CHI)-(AuNPs)-(CHI)-(CuPcS)]_2_ (solid line) and of the biosensor [(CHI)-(AuNPs)-(CHI)-(CuPcS)]_2_-Lac (dotted line), Figure S5: a) FTIR spectra of the platform [(CHI)-(AuNPs)-(CHI)-(CuPcS)]_2_ with increasing number of layers (n = 4,8,12,16,20,24) and b) correlation between the transmittance measured at 1033 cm^−1^ and the number of layers, Figure S6: CV curves registered in catechol 10^−4^ mol·L^−1^ in 0.01 M phosphate buffer pH 7 at a bare ITO electrode, Figure S7: CV registered in catechol 10^−4^ mol·L^−1^ in 0.01 M phosphate buffer pH 7 at [(CHI)-(AuNPs)-(CHI)-(CuPcS)]_2_-Lac electrode, where the enzyme was cross-linked by immersion in glutaraldehyde liquid (dotted line) and exposure to glutaraldehyde vapors (solid line).
