# Supplementary material for: Biosensors Platform Based on Chitosan/AuNPs/Phthalocyanine Composite Films for the Electrochemical Detection of Catechol. The Role of the Surface Structure
Source: Sensors (Basel). 2020 Apr 10;20(7):2152. doi: 10.3390/s20072152 (PMC7181025; doi:10.3390/s20072152)
Supplement: Supplementary file 1 [file sensors-20-02152-s001.pdf]

sensors

Supplementary Material

Biosensors Platform Based on Chitosan/AuNPs/Phthalocyanine Composite Films for the Electrochemical Detection of Catechol. The Role of the Surface Structure

Coral Salvo-Comino [1,2], Alfonso González-Gil [1], Javier Rodriguez-Valentin [1,3], Celia Garcia-Hernandez [1,2], Fernando Martin-Pedrosa [2,3], Cristina Garcia-Cabezon [2,3] and Maria Luz Rodriguez-Mendez [1,2,*]

[1] Group UVASENS, Escuela de Ingenierías Industriales, Universidad de Valladolid, Paseo del Cauce, 59, 47011 Valladolid, Spain; coraldeugena@gmail.com (C.S.-C.); alfonsogoninf@gmail.com (A.G.-G.); javrova@gmail.com (J.R.-V.); celiagarciahernandez@gmail.com (C.G.-H.)
[2] Bioeco UVA Research Institute, Universidad de Valladolid, 47011 Valladolid, Spain; fmp@eii.uva.es (F.M.-P.); crigar@eii.uva.es (C.G.-C.)
[3] Dpt. of Materials Science, Universidad de Valladolid, Paseo del Cauce, 59, 47011 Valladolid, Spain
* Correspondence: mluz@eii.uva.es; Tel.: +34-983-423540; Fax: +34-983-423310

Received: 16 March 2020; Accepted: 09 April 2020; Published: date

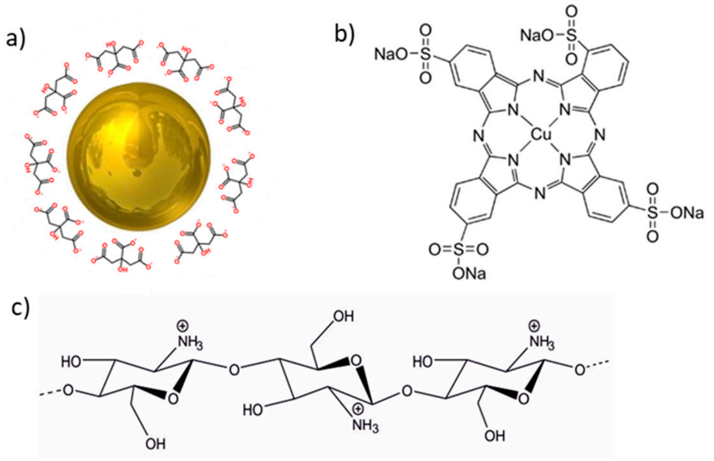

Figure S1. Materials used to form the sensing platforms: a) gold nanoparticles (AuNPs) capped with citrate; b) sulfonated copper phthalocyanine (CuPcS); and c) chitosan (CHI).

The AuNPs' size was recalculated following the Mie method, using the following equation:

$$d = \frac{ln(\frac{\lambda_{spr} - \lambda_0}{L1})}{L2}$$

where λ_{spr} is the surface plasmon resonance peak position. and λ_0=512 nm. L_1=6.53 nm and L_2=0.0216 nm-1, are theoretical parameters when d is known to be d>25 nm. The λ_{spr} value of 533 nm was determined from the absorbance spectrum shown below. The estimated diameter of the obtained AuNPs is 54.nm.

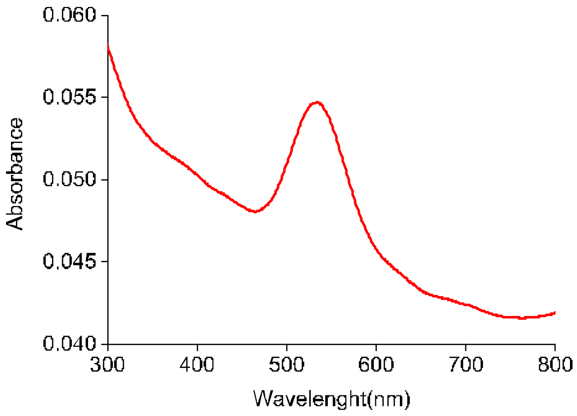

Figure S2. UV-Vis spectrum of AuNP solution.

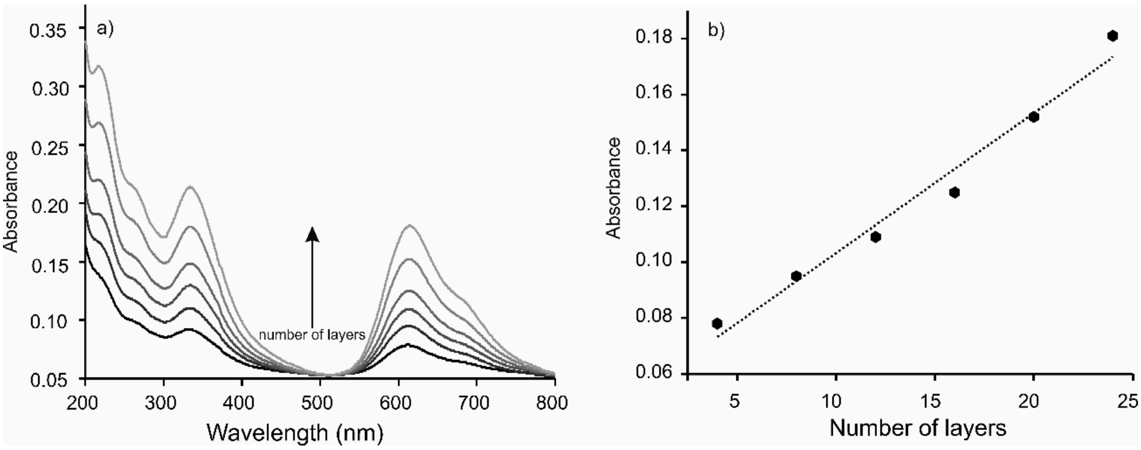

Figure S3. UV-Vis spectra of a) [(CHI)-(AuNPs)-(CHI)-(CuPcS)]$_2$ LbL films with increasing number of layers (n = 4,8,12,16,20); and b) correlation between the absorbance measured at 615 nm and the number of layers.

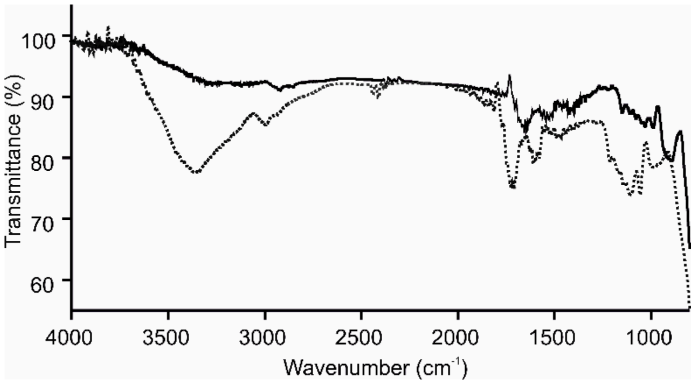

Figure S4. Comparison of the FTIR spectra of the LbL platform [(CHI)-(AuNPs)-(CHI)-(CuPcS)]$_2$ (solid line) and of the biosensor [(CHI)-(AuNPs)-(CHI)-(CuPcS)]$_2$-Lac (dotted line).

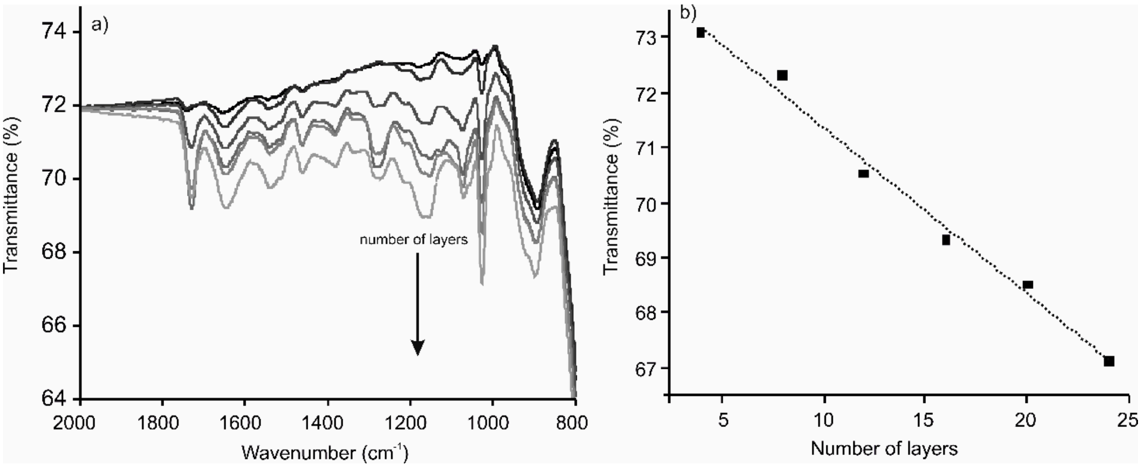

Figure S5. a) FTIR spectra of the platform [(CHI)-(AuNPs)-(CHI)-(CuPcS)]$_2$ with increasing number of layers (n = 4,8,12,16,20,24) and b) correlation between the transmittance measured at 1033 cm^{-1} and the number of layers.

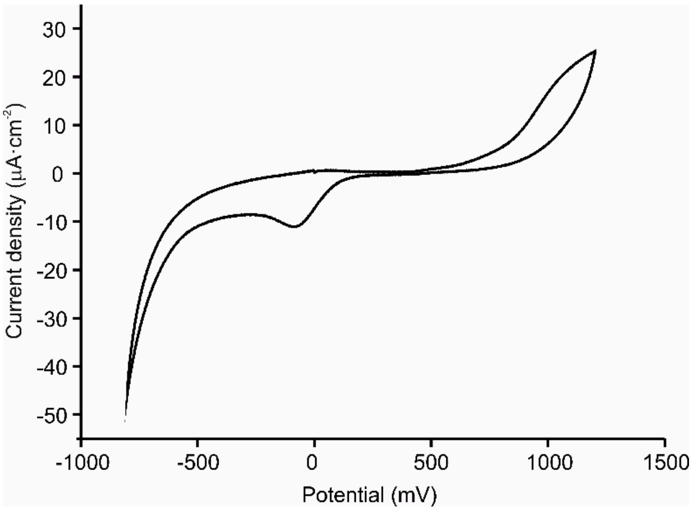

Figure S6. CV curves registered in catechol 10^{-4} mol·L^{-1} in 0.01 M phosphate buffer pH 7 at a bare ITO electrode**.**

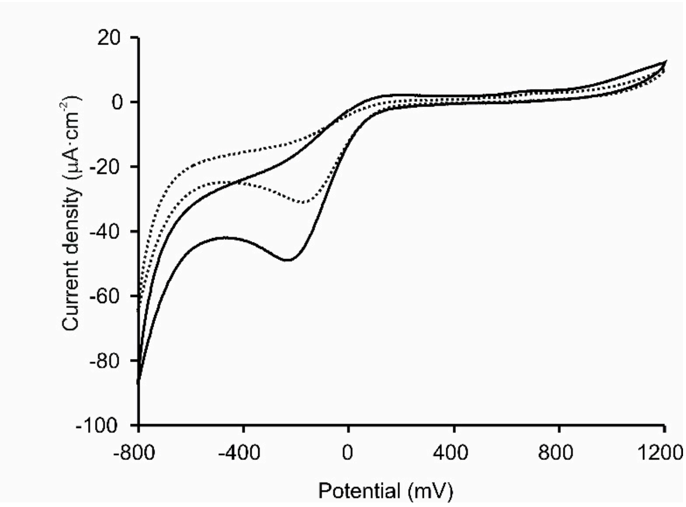

Figure S7. CV registered in catechol 10-4 mol·L-1 in 0.01 M phosphate buffer pH 7 at [(CHI)-(AuNPs)-(CHI)-(CuPcS)]2-Lac electrode, where the enzyme was cross-linked by immersion in glutaraldehyde liquid (dotted line) and exposure to glutaraldehyde vapors (solid line).

© 2020 by the authors. Submitted for possible open access publication under the terms and conditions of the Creative Commons Attribution (CC BY) license (http://creativecommons.org/licenses/by/4.0/).
